# Guidance for the practical management of the direct oral anticoagulants (DOACs) in VTE treatment

**DOI:** 10.1007/s11239-015-1310-7

**Published:** 2016-01-16

**Authors:** Allison E. Burnett, Charles E. Mahan, Sara R. Vazquez, Lynn B. Oertel, David A. Garcia, Jack Ansell

**Affiliations:** University of New Mexico Hospital Inpatient Antithrombosis Service, University of New Mexico College of Pharmacy, 2211 Lomas Blvd. NE, Albuquerque, NM 87106 USA; Presbyterian Healthcare Services, University of New Mexico College of Pharmacy, Albuquerque, NM USA; University of Utah Health Care Thrombosis Center, Salt Lake City, UT USA; Anticoagulation Management Service, Massachusetts General Hospital, Boston, MA USA; Division of Hematology, University of Washington School of Medicine, Seattle, WA USA; Hofstra North Shore/LIJ School of Medicine, Hempstead, NY USA

**Keywords:** DOACs, NOACs, Direct thrombin inhibitors, Factor Xa inhibitors, Antidotes, Care transitions, Bridging anticoagulation, Drug interactions

## Abstract

Venous thromboembolism (VTE) is a serious medical condition associated with significant morbidity and mortality, and an incidence that is expected to double in the next forty years. The advent of direct oral anticoagulants (DOACs) has catalyzed significant changes in the therapeutic landscape of VTE treatment. As such, it is imperative that clinicians become familiar with and appropriately implement new treatment paradigms. This manuscript, initiated by the Anticoagulation Forum, provides clinical guidance for VTE treatment with the DOACs. When possible, guidance statements are supported by existing published evidence and guidelines. In instances where evidence or guidelines are lacking, guidance statements represent the consensus opinion of all authors of this manuscript and are endorsed by the Board of Directors of the Anticoagulation Forum.

The authors of this manuscript first developed a list of pivotal practical questions related to real-world clinical scenarios involving the use of DOACs for VTE treatment. We then performed a PubMed search for topics and key words including, but not limited to, apixaban, antidote, bridging, cancer, care transitions, dabigatran, direct oral anticoagulant, deep vein thrombosis, edoxaban, interactions, measurement, perioperative, pregnancy, pulmonary embolism, reversal, rivaroxaban, switching, \thrombophilia, venous thromboembolism, and warfarin to answer these questions. Non- English publications and publications > 10 years old were excluded. In an effort to provide practical information about the use of DOACs for VTE treatment, answers to each question are provided in the form of guidance statements, with the intent of high utility and applicability for frontline clinicians across a multitude of care settings.

## Introduction

The availability of the new direct oral anticoagulants (DOACs) has significantly changed the therapeutic landscape of anticoagulation and these agents may eventually displace conventional VTE treatment with a rapid-acting parenteral anticoagulant overlapped with a vitamin K antagonist (e.g. warfarin) in appropriately selected patients. As a class, the DOACs exhibit comparable efficacy and a significantly lower bleeding risk compared to warfarin among patients with acute symptomatic VTE [1, 2]. For patients who need extended anticoagulation for secondary VTE prevention, the safety record of the DOACs is strong [3–5].

In this paper we will examine key questions pertaining to the practical management of DOACs for VTE treatment, summarize the evidence (where it exists) pertaining to those questions, and finally, provide guidance that may be applied to real-world practice by frontline clinicians.

## Methods

To provide guidance on the practical management of the DOACs, we first developed a number of pivotal practical questions that apply to DOACs as they might be used in the treatment of VTE. (Table 1). Questions were developed by consensus of the authors. The medical literature was reviewed using PubMed for topics and key words including, but not limited to, adherence, anticoagulant, apixaban, appropriate patient selection, bleed, bridging, care transitions, adherence, CYP, dabigatran, deep venous thrombosis (DVT), direct, edoxaban, education, follow-up, hemorrhage, initiation, interaction, measurement, monitoring, novel, oral, peri-operative, p-glycoprotein, practical management, prothrombin complex concentrate (PCC) pulmonary embolism (PE), reversal, rivaroxaban, safety, switching, target-specific, temporary interruption (TI), and venous thromboembolism (VTE). Non-English language publications and publications >10 years old were excluded. Guidance provided in this document is, whenever possible, based on the best available evidence. For some issues, however, published evidence is lacking. In all instances, guidance statements represent the consensus opinion(s) of all authors and are endorsed by the Anticoagulation Forum’s Board of Directors.

**Table 1:** Guidance questions to be considered

1. Which VTE patients are (and are not) good candidates for DOAC therapy?
2. How should DOACs be initiated for VTE treatment?
3. How should the anticoagulant activity of DOACs be measured?
4. How should VTE patients who require temporary interruption of DOAC therapy be managed?
5. How should patients with DOAC drug–drug interactions be managed?
6. How should patients transition between anticoagulants?
7. How should DOAC-associated bleeding be managed?
8. What is an appropriate care transitions and follow-up strategy for VTE patients on DOAC therapy?
9. How can patients enhance safety and efficacy of their DOAC therapy?

## Guidance

1. Which VTE patients are (and are not) good candidates for DOAC therapy?

The DOACs have been studied extensively in clinical trials and the results demonstrate they are at least as safe and effective as conventional treatment in the majority of typical VTE patients. However, many specific subgroups were excluded or underrepresented in these studies and the safety and efficacy of DOACs within these subgroups has yet to be established. The inclusion criteria for the VTE treatment trials included patients age ≥18 (no pediatric studies have been published) with an acute symptomatic proximal DVT and/or PE. Exclusion criteria varied slightly among the trials, but in general, patients were excluded if they had any of the following: need for thrombolytic therapy, another indication for anticoagulation, high risk of bleeding, clinically significant liver disease (acute or chronic hepatitis, cirrhosis, or alanine aminotransferase level greater than three times the upper limit of normal), creatinine clearance (CrCl) <30 mL/min (for apixaban the threshold was 25 mL/min), life expectancy of <3–6 months, aspirin use >100 mg/day, using interacting medications, uncontrolled hypertension, breastfeeding or pregnant or of childbearing potential without appropriate contraceptive measures [3–10]. Table 2 represents potential advantages and disadvantages of DOACs in comparison to conventional therapy that should be considered by both clinicians and patients before deciding on an anticoagulant regimen. Table 3 provides selection criteria for patients suitable for DOAC therapy. Table 4 provides further considerations regarding patient-controlled aspects, such as adherence, values and preferences as each of these will have a direct impact on outcomes with DOAC therapy.

**Table 2:** Potential advantages and disadvantages of DOACs compared to VKAs [119]

Advantages	Disadvantages
No routine monitoring	No reliable, readily available measurement assay
Improved safety profile	Dose reduction or avoidance in renal impairment and avoidance in moderate or severe hepatic impairment
Rapid onset (may preclude the need for induction or bridging therapy)	No specific antidote
Short half-life (advantageous for invasive procedures or in the setting of active bleed)	Short half-life (mandates strict adherence)
Fixed dosing	Less flexibility in dosing
Greater convenience, patient satisfaction and quality of life	Fewer studies and approved indications (e.g., contraindicated in mechanical valve replacement)
Potentially more cost-effective from health system perspective	Potentially higher drug acquisition costs for patients
Fewer drug, disease and diet interactions	DOAC drug interactions do exist that may preclude use

**Table 3:** DOAC patient selection criteria

Criteria for DOAC use	Comment(s)
Patient preference for and willingness to take DOAC	Patients should be presented will all therapeutic options and their respective perceived advantages and disadvantages (See Table 2)
No contraindication to DOAC therapy	E.g. pregnancy, breastfeeding, mechanical heart valve
Adequate organ function	Clinicians should regularly monitor renal function, particularly for DOACs with greater reliance on renal elimination (see Tables 5, 6 and 12) and, if there are other factors that may increase DOAC exposure (e.g. age, unavoidable use of concomitant p-gp/CYP3A4 inhibitors). Avoid in moderate or severe hepatic dysfunction
No significant drug–drug interactions	See Tables 13 and 14 for detailed guidance Patients taking *any* anticoagulant with antiplatelet agents or NSAIDs have a significantly higher risk of bleeding. To minimize bleeding, avoid these drug combinations when possible
No significant disease state interactions	VTE patients with a history of GI bleeding or at risk for GI bleeding may be better candidates for warfarin, apixaban, or edoxaban, as there may be a higher risk of bleeding or GI adverse effects with dabigatran and rivaroxaban
Highly likely to be adherent with DOAC therapy and follow-up plan	See Table 4 for further details
Confirmed ability to obtain DOAC on a longitudinal basis from a financial, insurance coverage and retail availability standpoint	The drug costs of DOACs may be prohibitive for some patients, as compared with generic warfarin plus laboratory monitoring There are patient assistance programs available via the pharmaceutical companies, and this should be arranged prior to prescribing

**Table 4:** Patient adherence assessments when choosing anticoagulant therapies [118–123]

Taking medications	**How often does the patient miss or forget to take doses of their medication(s)?** • If a warfarin patient frequently misses doses, switching to a shorter half-life DOAC may more rapidly predispose the patient to risk of thrombosis • Often, a subtherapeutic INR is a reliable indicator to the clinician and patient that warfarin doses have been missed • Without the requirement for laboratory monitoring with the DOACs, there is no such alert to indicate opportunities to improve adherence
	**Is a once-daily or a twice-daily medication dosing frequency preferred?** • If patient is adherent with other twice daily medications, any of the DOACs may be appropriate • Conversely, if patient prefers once daily medications, rivaroxaban or edoxaban may be preferred
Laboratory monitoring	**Is laboratory access difficult?** • Patients with transportation challenges, difficult venous access, inflexible work or school schedules or other reasons for difficulty complying with INR monitoring may significantly benefit from DOAC therapy • Clinicians should remind DOAC patients that renal function and a complete blood count should be monitored at least annually or more frequently as the clinical situation dictates
Health care responsibility	**Is the patient reliable to notify health care providers about changes to health and pertinent medical issues?** • It is important for the patient to make all health care providers aware he or she is taking an anticoagulant medication, as this information will aid in: – design of peri-procedural anticoagulation plans – addressing medication interactions – consideration of other health status changes • Patients who may be unreliable to report pertinent issues to the clinician may be better suited to warfarin so that at least some of these may be uncovered during INR follow-up • DOAC patients and their clinicians may elect to interact via clinic visit, phone, or electronic media at a regular interval

*INR* International normalized ratio, *DOAC* direct oral anticoagulant

*Pregnancy and breastfeeding*

Animal studies of dabigatran and rivaroxaban demonstrated pregnancy loss and fetal harm [11, 12], and one study demonstrated that dabigatran does cross the human placenta [13]. A case report of maternal rivaroxaban use during weeks 1–19 of pregnancy (when pregnancy discovered at week 19, the patient was switched to enoxaparin) resulted in a full-term, low growth percentile, otherwise healthy infant [14]. Apixaban has no human data in pregnancy, but showed no maternal or fetal harm in animal studies [15]. Edoxaban animal studies demonstrated no fetal harm. The edoxaban VTE treatment trial reported 10 pregnancies, with edoxaban exposure during the first 6 weeks of gestation (4 full-term births, 2 pre-term births, 1 first-trimester spontaneous abortion, and 3 elective pregnancy terminations) [16]. It is unknown whether any of the DOACs are excreted in breast milk. Because of the potential for infant harm, a decision should be made to either avoid breastfeeding or use an alternative anticoagulant, such as warfarin, in these women.

*Body weight extremes*

Patients at extremes of weight represented a very small proportion of subjects in DOAC VTE treatment trials. [3–10]. The mean weight was around 84 kg, with the majority of patients weighing between 60 and 100 kg. Underweight patients (<50–60 kg) comprised 2–13 % of the study populations and roughly 14–19 % of patients were >100 kg. Approximately 30 % of patients in the EINSTEIN, AMPLIFY and RE-COVER studies had a BMI ≥ 30 kg/m^2^, and in the AMPLIFY and RE-COVER studies, only 12 % of subjects had a BMI ≥ 35 kg/m^2^. Based on very limited data, extremes of weight do not appear to affect peak concentrations or bioavailability of dabigatran [17]. The pharmacokinetics and pharmacodynamics of factor Xa inhibitors may be affected by weight [10, 15, 18–20], but the clinical impact of these effects remains unknown. Pending further evidence in patients at extremes of weight (e.g., <50 kg, >120 kg or BMI ≥ 35 kg/m^2^) it is advisable to limit DOAC use to situations where vitamin K antagonists cannot be used.

*Thrombophilia*

Patients with thrombophilias represented 2–18 % of DOAC VTE clinical trial populations [3–9]. A posthoc subgroup analysis of thrombophilia patients within the RE-MEDY trial was recently presented [21]. Results showed that the frequencies of VTE-related death and PE did not differ between dabigatran and warfarin patients. The authors concluded that dabigatran’s efficacy in preventing recurrent VTE is not influenced by the presence of thrombophilia. Conversely, six cases citing possible failure of rivaroxaban or dabigatran to prevent thrombosis in patients with antiphospholipid antibody syndrome were recently published [22, 23]. While it is possible the DOACs may be a viable option for VTE treatment in patients with weaker underlying thrombophilias (e.g., heterozygous Factor V Leiden), caution or avoidance, especially in highly pro-thrombotic states such as antiphospholipid antibody syndrome or heparin-induced thrombocytopenia, is suggested until further evidence becomes available.

*Cancer*

Four meta-analyses of DOAC VTE clinical trials including approximately 1000 cancer patients (patients with a history of cancer or some with active cancer) demonstrated similar efficacy and safety for the DOACs compared to conventional therapy of a vitamin K antagonist overlapped with LMWH [24–27]. Previous trials, which included approximately 2000 patients with active cancer (many in advanced stages), indicate that vitamin K antagonists are inferior to long-term LMWH monotherapy for treatment of cancer-related VTE [28–31]. While most evidence to date is with dalteparin, the recent CATCH study [32] showing a trend (*p* = 0.07) towards superiority of tinzaparin over warfarin for prevention of recurrent symptomatic DVT and reduction in clinically relevant non-major bleeding suggests this may be a class effect of the LMWHs. Whether DOACs convey similar benefit as LMWH monotherapy for VTE treatment in cancer patients remains unknown. Data from head-to-head randomized controlled trials or robust comparative effectiveness studies is needed and future research in this area is encouraged. Until then, among patients with cancer-associated VTE, long-term LMWH is the preferred first-line therapy for anticoagulant treatment (see chapter by Khorana et al.). However, for those patients who cannot (or will not) use long term LMWH, either a DOAC or VKA could be prescribed as a second-line option. Given their improved safety profile compared to warfarin, DOACs may well be preferred in these instances, particularly among patients with a perceived increased risk for bleeding. However, it is important to emphasize the lack of experience with DOACs compared to warfarin in cancer patients who may have profound thrombocytopenia and other clinical challenges pertaining to anticoagulation. The lack of readily available measurement assays for DOACs may be particularly problematic in the setting of drug interactions, nephrotoxic chemotherapy, and potential disruption in absorption due to short gut or malnutrition, common issues in a cancer population.

*History of bleeding*

Much of the available data on DOACs and gastrointestinal (GI) bleeding is from atrial fibrillation trials, which generally consisted of older patients with more comorbidities than the VTE treatment populations. In a real- world study of Medicare claims data among new users of dabigatran or warfarin for non-valvular atrial fibrillation [33], there was a 28 % overall increased risk for gastrointestinal bleeding among dabigatran patients compared to warfarin patients. This was most pronounced in women ≥75 years of age (HR 1.5; 95 % CI 1.2–1.88), men ≥85 years of age (HR 1.55; 95 % CI 1.04–2.32) and in patients receiving the higher dose of 150 mg twice daily (HR 1.51; 95 % CI 1.32–1.73). A meta-analysis of 4 dabigatran trials of both NVAF and VTE treatment reported a 41 % increase in the risk of GI bleeding with dabigatran [34]. In the individual DOAC VTE treatment trials [3–10], GI bleeding event rates were too low to draw definite conclusions (dabigatran and rivaroxaban numerically higher rates of GI bleeding, apixaban and edoxaban numerically lower rate of GI bleeding) compared to conventional anticoagulation therapy. A meta-analysis of data from 11 phase-3 DOAC NVAF or VTE treatment trials found no significant difference in major gastrointestinal bleeding between DOACs and warfarin (2.09 vs. 1.7 %; RR 0.94; 95 % CI 0.75–1.99; *p* = 0.62, I^2^ 71 %) [35]. Even so, careful consideration should be exercised in regards to DOAC use in patients with a history of gastrointestinal bleeding.

Intracranial hemorrhage (ICH) is the most feared complication of anticoagulant therapy. A significant advance with DOAC therapy over warfarin has been a reduction in the rates of ICH in atrial fibrillation. Numerically lower rates of both ICH and fatal bleeding were seen in all DOAC arms of the VTE trials [3–10], with the exception of intracranial hemorrhage in the EINSTEIN-DVT trial (2 events in the rivaroxaban arm vs. none in the warfarin arm) [4]. A systematic review and meta-analysis of 12 randomized controlled trials including over 100,000 patients with either NVAF or VTE showed that DOACs are associated with less major bleeding, fatal bleeding, intracranial bleeding, clinically relevant non-major bleeding, and total bleeding compared to warfarin [35]. This provides a compelling argument to favor these agents over conventional therapy for VTE treatment whenever possible.

### **Guidance statement**

*DOACs are suggested as an alternative to conventional therapy for VTE treatment in patients who meet appropriate patient selection criteria. For all other patients, we suggest VTE treatment with conventional therapy. Until further data are available, we suggest avoiding DOACs for VTE in patients with antiphospholipid antibody syndrome and patients at extremes of weight. LMWH monotherapy remains first line for patients with cancer-related VTE, but DOACs may be considered in select patients unwilling or unable to receive subcutaneous injections.*

2. How should DOACs be initiated for VTE treatment?

Before prescribing a DOAC, a thorough evaluation should be conducted to ensure the patient is a good candidate for DOAC therapy, as detailed in Tables 3 and 4. Baseline labs should be performed, including serum creatinine, liver function tests, complete blood count, and coagulation assays such as aPTT and PT to ensure adequate organ function and rule out coagulopathy. In general, DOAC therapy should not be initiated in patients presenting with extensive VTE if there is potential need for an invasive procedure, such as thrombolysis or thrombectomy. Instead, preference should be given to a shorter-acting, reversible agent such as unfractionated heparin until no further immediate procedures are needed. Clinicians should consider characteristics of the individual agents when selecting which DOAC to initiate (detailed in Table 5). In addition, concomitant drug therapies and comorbidities should also be accounted for in DOAC dose management as detailed in Table 6. In clinical trials of edoxaban and dabigatran [6, 10] initial treatment consisted of open-label parenteral anticoagulation (median of 9 and 7 days in the dabigatran and edoxaban trials, respectively) overlapped with warfarin titrated to an INR of 2–3 in the control arm or overlapped with warfarin-placebo titrated to a sham INR in the intervention arms. Concomitant administration of a parenteral anticoagulant and a DOAC was not employed in either of these trials, as that would likely lead to excessive anticoagulation based on the rapid onset of the DOACs. Dabigatran was initiated at 150 mg BID. Edoxaban was initiated at 60 mg once daily, with a dose reduction to 30 mg once daily in patients with a creatinine clearance of 30–50 mL/min or a body weight of 60 kg or less or in patients who were receiving concomitant treatment with potent P-glycoprotein inhibitors. Package labelling for dabigatran and edoxaban also indicates the required 5–10 days of parenteral anticoagulation prior to their initiation for acute VTE, which closely approximates the conventional approach to VTE treatment.

**Table 5:** Drug characteristics to consider when deciding which DOAC to prescribe for VTE [3–12, 15, 16]

DOAC	Parenteral lead-in	Single-drug approach	Switch or dose de-escalation	Dosing frequency	Renal elimination	Potential for increased adverse effects
Dabigatran	√		√	BID	++++	MI, GIB, dyspepsia
Rivaroxaban		√	√	BID × 21 days, then once daily	++	GIB
Apixaban		√	√	BID	+	N/A
Edoxaban	√		√	Once daily	++	N/A

*BID* twice daily, *GIB* gastrointestinal bleed, *MI* myocardial infarction

**Table 6:** Dosing of DOACs for VTE treatment [3–12, 15, 16]

	Dabigatran	Rivaroxaban	Apixaban	Edoxaban
Acute VTE	150 mg BID after ≥5 days of parenteral anticoagulation	15 mg BID with food × 3 weeks then 20 mg once daily with food	10 mg BID for 7 days, then 5 mg BID	60 mg once daily after ≥5 days of parenteral anticoagulation
Prevention of VTE recurrence	No dose adjustment	No dose adjustment	Decrease to 2.5 mg BID after at least 6 months of therapeutic anticoagulation	Not studied
Dosage adjustments and/or thresholds for avoidance	Any P-gp *inducer*: avoid concurrent use Any P-gp *inhibitor* with CrCl <50 mL/min: avoid concurrent use CrCl <30 mL/min: avoid use	CrCl < 30 mL/min: avoid use Dual strong CYP3A4 and P-gp *inhibitors or inducers*: avoid use	Dual strong CYP3A4 and P-gp *inducers*: avoid use Dual strong CYP3A4 and P-gp *inhibitors*: If dose >2.5 mg BID, decrease dose by 50 % If already taking 2.5 mg BID and dual strong CYP3A4 and P-gp *inhibitor*: avoid use No dose adjustment for renal impairment provided	30 mg once daily if any of the following: CrCl 15–50 mL/min Weight < 60 kg Concomitant P-gp *inhibitor* CrCl < 15 mL/min: avoid use

*DOAC* direct-acting oral anticoagulant, *VTE* venous thromboembolism, *BID* twice daily, *P-gp* P-glycoprotein, *CrCl* creatinine clearance, *CYP3A4* cytochrome P-450 3A4

For patients with acute VTE selected for treatment with edoxaban or dabigatran, for lead-in therapy we suggest use of subcutaneous (SC) anticoagulants LMWH or fondaparinux over unfractionated heparin (UFH) when possible due to improved safety and efficacy [36, 37] and facilitation of outpatient therapy in eligible patients. (See care transitions section for more details). When switching from lead-in parenteral therapy within the acute VTE treatment phase, edoxaban or dabigatran should be initiated at the time that a heparin infusion is discontinued or the time the next dose of SC anticoagulant is due.

In clinical trials of apixaban [5] and rivaroxaban [4, 8], a single-drug approach was employed without parenteral anticoagulation. A higher dose was used in the initial period followed by a dose reduction(s). Apixaban was initiated with 10 mg BID for the first 7 days and reduced to 5 mg BID thereafter. Rivaroxaban was initiated at 15 mg BID for 21 days followed by 20 mg once daily. Less than 2 % of patients in apixaban and rivaroxaban VTE treatment trials received >2 days of parenteral anticoagulation before randomization which reinforces that these agents can be safely used as an oral, single-drug strategy for VTE treatment. Rivaroxaban and apixaban monotherapy should be initiated as soon as it is determined that no invasive procedures are needed. If the patient has been receiving empiric or temporary UFH or SC anticoagulant therapy for acute treatment of VTE, apixaban or rivaroxaban should be initiated at the time that the heparin infusion is discontinued or at the time the next dose of SC anticoagulant is due.

### **Guidance statement**

*We suggest that a thorough patient evaluation be conducted prior to DOAC initiation which should include assessment of baseline laboratory values, concomitant drug therapies, and comorbidities. We do not recommend initial DOAC therapy in patients who are hospitalized with extensive DVT or who have PE with hemodynamic instability in whom thrombolysis or thrombectomy may be indicated. We suggest that the unique characteristics of each DOAC, their distinct dosing for VTE treatment, and patient preferences should be considered when selecting a DOAC for VTE treatment.*

3. How should the anticoagulant activity of DOACs be measured?

**Fig. 1:**
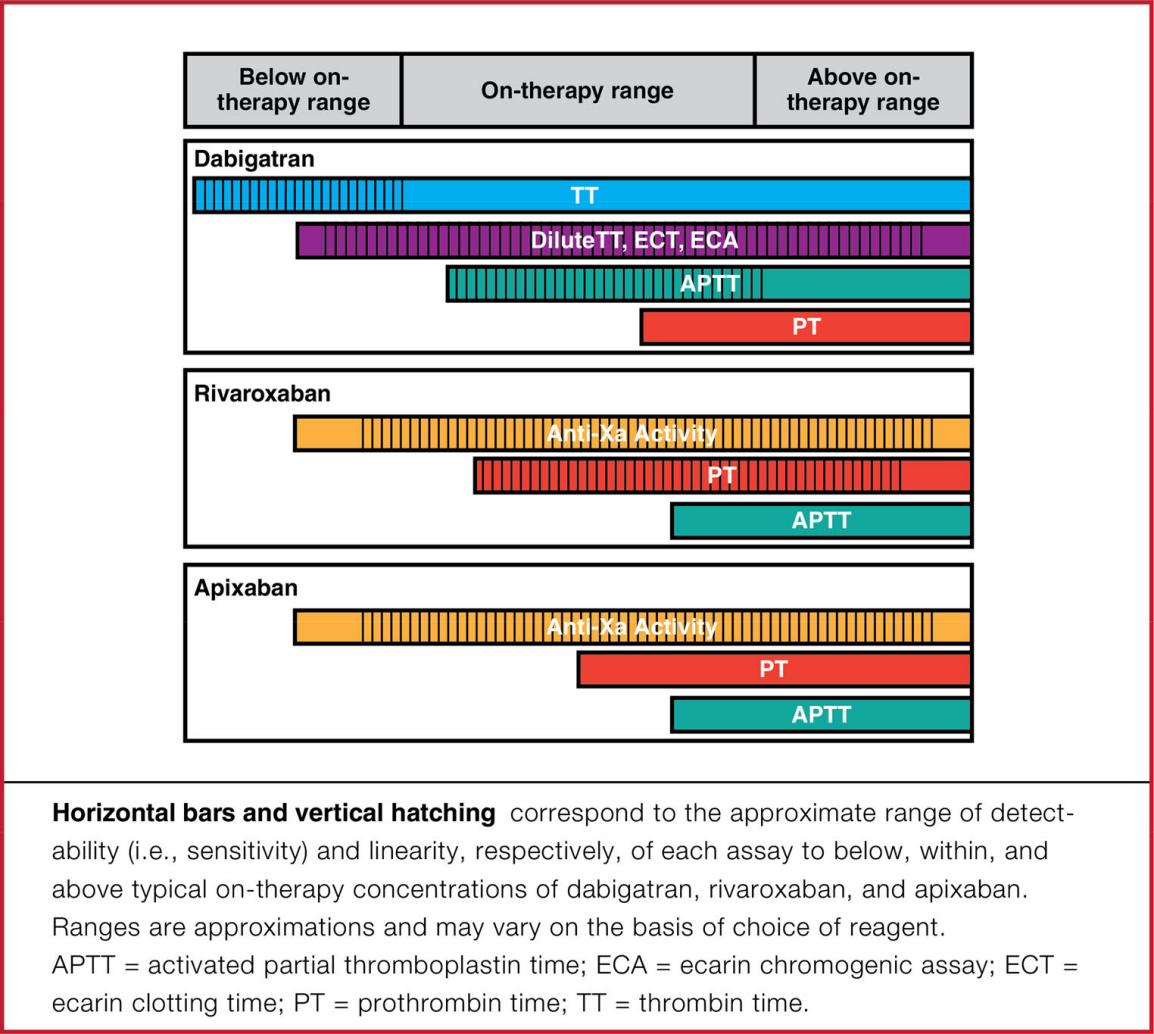
Linearity and specificity of coagulation assays for measurement of DOACs [40]. Reproduced with permission from Cuker et al. [40]

The specificity, predictability and wide therapeutic index of the DOACs allow for fixed dosing without a need for routine monitoring. However, there are instances during which measurement of DOAC activity would be useful to direct therapy and inform long-term treatment decisions (Table 7) [38–40]. When these situations occur, clinicians need to be familiar with the role, limitations and local availability of various coagulation assays as they relate to DOACs (Tables 7, 8; Fig. 1).

**Table 7:** Potential indications for DOAC measurement [38–40]

Detection of clinically relevant levels	Detection of expected on-therapy levels	Detection of excessive levels
Urgent or emergent invasive procedure	Assessing adherence	Hemorrhage
Neuraxial anesthesia	Breakthrough thrombosis	Diminished/changing renal function
Major trauma		Hepatic impairment
Potential thrombolysis in acute thromboembolism		Accidental or intended overdose
Hemorrhage		Drug interactions
		Advanced age

**Table 8:** Suggestions for laboratory measurement of DOACs [40]

Clinical objective						
Drug	Determine if clinically relevant below on-therapy drug levels are present		Estimate drug levels within on-therapy range		Determine if above on-therapy drug levels are present	
	Suggested test	Interpretation	Suggested test	Interpretation	Suggested test	Interpretation
Dabigatran	TT	Normal TT likely excludes clinically relevant drug levels	Dilute TT, ECA, ECT		aPTT, dilute TT, ECA, ECT	Normal aPTT likely excludes excess drug levels; only dilute TT, ECA, and ECT are suitable for quantitation
Rivaroxaban	Anti-Xa	Normal anti-Xa activity likely excludes clinically relevant drug levels	Anti-Xa		Anti-Xa, PT	Normal PT likely excludes excess drug levels; only Anti-Xa is suitable for quantitation
Apixaban	Anti-Xa	Normal anti-Xa activity likely excludes clinically relevant drug levels	Anti-Xa		Anti-Xa	Normal PT may not exclude excess drug levels; only Anti-Xa is suitable for quantitation
Edoxaban	Anti-Xa	Normal anti-Xa activity likely excludes clinically relevant drug levels	Anti-Xa		Anti-Xa, PT	Normal PT likely excludes excess drug levels; only Anti-Xa is suitable for quantitation

*aPTT* Activated partial thromboplastin time, *ECA* ecarin chromogenic assay, *ECT* ecarin clotting time, *PT* prothrombin time, *TT* thrombin time, need permission from Cuker et al. JACC 2014 [40]

The INR does not vary significantly from hour to hour due to the long half-life of warfarin and the timing of INR in relation to the last warfarin dose is not important. In contrast, the timing of last DOAC dose relative to the coagulation assay is important for interpretation given the relatively short half-life of the DOACs [39]. Most scenarios that would trigger laboratory testing for DOACs are urgent (e.g. bleeding or thrombosis) thus lab results will often be random out of necessity. In the bleeding patient, it is likely sufficient to have a rapidly available quantitative test that will reliably determine whether DOAC is present in measurable quantities (yes or no). In the setting of thrombosis or suspected treatment failure, the ideal test would indicate not only whether drug was present but also if the concentration was consistent with observed on-treatment levels. In the event of concern for DOAC accumulation due to renal insufficiency or drug interactions, trough levels are preferred [39]. For detailed information of the impact of individual DOACs on various anticoagulant assays, please refer to the pharmacology chapter of this compendium by Nutescu et al.

A systematic review regarding laboratory measurement of OAC activity was recently published and provides support for following guidance statements: [40]

### **Guidance statement**

*We suggest that clinicians do not routinely measure DOAC activity. If measurement of a DOAC is indicated, we suggest that clinicians use assays that are validated either locally or in a reference laboratory and that are readily available. The chosen assay should be suitable for the DOAC being used, as well as for the indication for measurement, as detailed in Table* 8.

4. How should VTE patients who require temporary interruption (TI) of DOAC therapy be managed?

*Use of bridge therapy*

Approximately 10 % of patients require temporary interruption (TI) of their anticoagulant for a procedure on an annual basis [41] with additional patients requiring interruption due to bleeding or other non-bleeding adverse events [42–44]. Relatively little direct medical literature exists on studies in the VTE treatment population and TI of DOACs; however, some information may be extrapolated from data in other populations as well as guidelines and other practical recommendations. [42–44]. In the RELY and the ROCKET atrial fibrillation (AF) trials, 25 and 33 % of enrolled participants underwent one or more TI during the study period with 17 and 8.2 % utilizing bridging with unfractionated heparin (UFH)/low molecular weight heparin (LMWH) therapy for dabigatran 150 mg and rivaroxaban, respectively [42, 43]. In the RELY trial, there were numerically more bleeding events among dabigatran patients who received bridging therapy compared to warfarin patients receiving bridging therapy (295 for dabigatran 150 mg BID vs. 276 for warfarin). There was no significant difference in the incidence of perioperative major bleeding (4.6 vs. 5.1 %) or the composite of cardiovascular death, ischemic stroke, and non-central nervous system and pulmonary embolism (1.2 vs. 1.5 %) for warfarin and dabigatran 150 mg BID, respectively. Other bleeding outcomes, including fatal bleeding, bleeding requiring reoperation or transfusion of red blood cells, and minor bleeding were also similar between groups [42]. For rivaroxaban, perioperative major bleeding (0.99 %/30 days vs. 0.79 %/30 days) and the composite of stroke/systemic embolism/myocardial infarction/death (0.66 %/30 days vs. 0.95 %/30 days) were not significantly different for the rivaroxaban and warfarin groups that received bridging therapy, respectively. Overall in the ROCKET AF trial in TI patients, there was numerically higher major/non-major clinically relevant bleeding for those who received bridging therapy versus those who did not (4.83 vs. 3.02 %) [43]. The data from these subanalyses suggests that bridging therapy with LMWH/UFH should be minimized or avoided in DOAC patients. The pharmacokinetic similarities of these two anticoagulant classes further support avoidance of overlapping therapies to prevent over anticoagulation.

*Managing DOAC interruptions for invasive procedures*

Determining the optimal approach to management of DOACs around elective invasive procedures involves addressing a few key clinical questions. For elective procedures, clinicians should first consider whether the procedure can be delayed until a time that the patient may not require a DOAC or is at least several months after the index event, since the risk of recurrent VTE is highest during the first 3 months. For patients who require long-term anticoagulation or in whom the invasive procedure cannot be delayed, the next step is to determine whether procedure-related bleeding risk is sufficiently high to warrant DOAC interruption. Some procedures, such as simple dental extractions, minor dermatologic procedures or cataract surgery, pose minimal bleed risk and do not require interruption of anticoagulation. Table 9 provides a list of procedures categorized by bleed risk. Table 10 lists additional characteristics that may predispose patients to bleeding.

**Table 9:** Procedural bleed risk [41, 46, 47]

MINIMAL bleed risk procedures that may not require interruption of anticoagulant therapy	LOW bleeding risk procedures requiring interruption of anticoagulant therapy	HIGH bleeding risk procedures requiring interruption of anticoagulant therapy
Central venous catheter removal Dental procedures Extraction of 1–2 teeth Periodontal surgery Incision of abscess Implant positioning Endoscopy without surgery Ophthalmology Cataract or glaucoma intervention Superficial surgery Abscess incision Small dermatology excisions	Abdominal hernia repair Abdominal hysterectomy Carpal tunnel repair Cholecystectomy Dental procedures Extraction of 3 or more teeth Dilatation and curettage Electrophysiological study or radiofrequency catheter ablation for supraventricular tachycardia (including left-sided ablation via single transseptal puncture) Endoscopy with biopsy or tissue removal Gastrointestinal endoscopy ± biopsy, enteroscopy, biliary/pancreatic stent without sphincterotomy, endosonography without fine-needle aspiration Hemorrhoidal surgery Hydrocele repair Non-coronary angiography bronchoscopy ± biopsy Ophthalmology Non-cataract eye surgery Prostate or bladder biopsy Shoulder/foot/hand surgery and arthroscopy	Any major surgery (procedure duration >45 min) Abdominal and gastrointestinal surgeries Bowel resection Abdominal aortic aneurysm repair Breast cancer surgery Cardiac surgeries Coronary artery bypass Heart valve replacement Cardiac procedures Complex left-sided ablation (pulmonary vein isolation; VT ablation) Implantation of a pacemaker, implantable cardioverter defibrillator, or cardiac resynchronization therapy defibrillator Endoscopically guided fine-needle aspiration Head or neck surgery Hepatic surgeries and procedures including liver biopsy Major orthopedic surgery Joint replacement/arthoplasty Prosthetic revision Miscellaneous surgeries and procedures Biliary sphincterectomy PEG placement Pneumatic dilatation Polypectomy Variceal treatment Neurosurgeries Plastic surgery Major reconstructive surgery Spinal surgeries or procedures Spinal or epidural anaesthesia Laminectomy Lumbar diagnostic puncture Splenic surgeries or procedures Thoracic surgery Urologic surgeries or procedures Kidney biopsy Bladder resection Nephrectomy Transurethral prostate resection Urologic cancer surgery or tumor ablation Vascular and general surgeries

**Table 10:** Patient-specific risk factors for bleeding [36, 124, 125]

General risk factors	Medical patient risk factors
Active or metastatic cancer Age (e.g. >65 years) Anemia Comorbidity and reduced functional capacity Concomitant medications such as NSAIDs, antiplatelets or other anticoagulants administered possibly in a transition period Diabetes Alcohol abuse Frequent falls Hepatic or renal dysfunction History of bleeding complications Previous stroke Recent surgery Thrombocytopenia	Age—increasing Active cancer Anemia and other blood dyscrasias Current liver disease Central venous catheter placement History of bleeding Hospital stay of ≥3 days ICU/CCU stay Male gender Previous or active gastroduodenal ulcer Thromboembolic stroke Recent re-hospitalization Renal failure Rheumatic disease

*NSAIDs* Nonsteroidal anti-inflammatory drugs, *ICU* intensive care unit, *CCU* cardiac care unit

When DOAC interruption is necessary, the cessation and resumption of the DOAC around the elective procedure is determined according to bleeding risk, renal function, and DOAC half-life (t_1/2_) (Table 11). The half-life of a drug is the time for the blood plasma concentration of a substance to reach one-half of its steady-state value as a result of elimination processes. It requires five half-lives to eliminate >95 % of a therapeutic drug concentration. When pathways of elimination are diminished (e.g. renal impairment), it will require more time to clear the drug and the half-life will increase. Among hospitalized VTE patients who develop acute kidney injury (AKI), the DOAC t_1/2_ may become significantly prolonged.

**Table 11:** Cessation and resumption of DOAC for TI^a^ [46, 47, 126, 127]

Cessation^b^				Resumption	
Renal function^c^ (mL/min)	Estimated half-life^d^ (hours)	Low bleeding risk surgery^e^ (allow 2–3 t_1/2_ between last dose and surgery)	High bleeding risk surgery^f^ (allow 4–5 t_1/2_ between last dose and surgery)	Low bleed risk	High bleed risk
Dabigatran (BID dosing)				1 day after procedure (~24 h post-op)	2-3 days after procedure^g^ (~48–72 h post-op)
CrCl > 80	t_1/2_ ~ 14	Hold time: 28–42 h # doses to hold: 2	Hold time: 56–70 h # doses to hold: 5–6		
CrCl > 50–79	t_1/2_ ~ 17	Hold time: 34–51 h # doses to hold: 3–4	Hold time: 68–85 h # doses to hold: 6–7		
CrCl 30–49	t_1/2_ ~ 19	Hold time: 38–57 h # doses to hold: 4–5	Hold time: 76–95 h # doses to hold: 7–8		
CrCl 15–29	t_1/2_ ~ 28	Hold time: 56–84 h # doses to hold: 5–7	Hold time: 112–140 h # doses to hold: 9–12		
CrCl < 15^h^	Unknown	Hold until resolved (e.g. if acute kidney injury) or consider transition to warfarin or UFH			
Rivaroxaban (Once daily dosing)					
CrCl > 80	t_1/2_ ~ 8	Hold time: 16–24 h # doses to hold: 1	Hold time: 32–40 h # doses to hold: 2		
CrCl > 30–79	t_1/2_ ~ 9	Hold time: 18–27 h # doses to hold: 1	Hold time: 36–45 h # doses to hold: 2		
CrCl 15–29	t_1/2_ ~ 10	Hold time: 20–30 h # doses to hold: 1–2	Hold time: 40–50 h # doses to hold: 2–3		
CrCl < 15^h^	Unknown	Hold until resolved (e.g. if acute kidney injury) or consider transition to warfarin or UFH			
Apixaban (BID dosing)					
CrCl > 50	t_1/2_ ~ 7–8	Hold time: 14–24 h # doses to hold: 2	Hold time: 28–40 h # doses to hold: 4		
CrCl 15–49	t_1/2_ ~ 17–18	Hold time: 34–54 h # doses to hold: 3–4	Hold time: 68–90 h # doses to hold: 6–7		
CrCl < 15^h^	Unknown	Hold until resolved (e.g. if acute kidney injury) or consider transition to warfarin or UFH			
Edoxaban (Once daily dosing)					
CrCl > 50	t_1/2_ ~ 8–9	Hold time: 16–27 h # doses to hold: 1	Hold time: 32–45 h # doses to hold: 2		
CrCl 30–49	t_1/2_ ~ 9–10	Hold time: 18–30 h # doses to hold: 1	Hold time: 36–50 h # doses to hold: 2		
CrCl 15–29	t_1/2_ ~ 17	Hold time: 34–51 h # doses to hold: 2	Hold time: 68–85 h # doses to hold: 3–4		
CrCl < 15^h^	Unknown	Hold until resolved (e.g. if acute kidney injury) or consider transition to warfarin or UFH			

^a^Applies to both elective procedures and procedures among hospitalized patients on DOAC treatment

^b^Consider earlier cessation of DOAC for patients with additional bleed risk factors listed in Table 10

^c^CrCl calculated using Cockroft–Gault method and actual body weight (ABW)

^d^Estimated t_1/2_ based on renal clearance

^e^Aiming for mild to moderate residual anticoagulant effect at surgery (12–25 %)

^f^Aiming for no or minimal residual anticoagulant effect (3–6 %) at surgery

^g^For patients at high risk for thromboembolism and bleeding after surgery, consider administering a prophylactic dose of anticoagulant on the first postoperative day. If the patient tolerates this, they may then be increased to treatment doses at 48–72 h post-procedure

^h^Consider laboratory measurement with appropriate assay to determine when it is safe to proceed with surgery

For urgent or emergent procedures, determination of time of last ingestion and rapid assessment of residual anticoagulant effect should be performed with an appropriate assay if possible before proceeding with invasive interventions. In deciding whether an urgent/emergent procedure should be delayed until after an appropriate amount of time has elapsed since the last administration of the DOAC, the increased risk of bleeding should be weighed against the urgency of the procedure.

Once hemostasis is achieved, the DOAC should be resumed approximately 24 h post-operatively in low bleed risk situations, and this should be delayed to 48–72 h in high bleed risk patients (Tables 9, 10, and 11). VTE prophylaxis with UFH, LMWH or DOAC may be employed, if necessary, until therapeutic doses of DOAC are resumed. If the risk of bleeding precludes even prophylactic-dose anticoagulation from being given, mechanical VTE measures should be considered. In situations where a patient cannot tolerate oral therapy post-operatively, apixaban or rivaroxaban may be administered via NG or a parenteral agent may be utilized until the DOAC can be administered. In post-operative patients with ongoing epidural anesthesia, DOACs should be avoided. Guidelines regarding neuraxial anesthesia and anticoagulants set forth by the American Society of Regional Anesthesia and Pain Medicine (ASRA) [45] should be strictly followed to avoid spinal or epidural hematoma. Only anticoagulants endorsed by ASRA should be utilized while the epidural remains in place.

Several guidelines and reviews pertaining to perioperative management of anticoagulants have been published and form the basis for our guidance statements [41, 46, 47].

### **Guidance statement**

*For VTE patients on DOAC therapy requiring TI for an invasive procedure, we suggest a carefully constructed, thoughtful approach that emphasizes communication between the provider managing the DOAC therapy, the clinician performing the procedure, and the patient and/or caregiver about the management of the DOAC. If TI is deemed necessary, we suggest that clinicians consider the patient’s renal function, the DOAC t*_*1/2*_* and the associated bleeding risk when determining timing of cessation and resumption of the DOAC. We suggest avoiding routine use of bridge therapy during DOAC interruption.*

5. How should patients with DOAC drug–drug interactions be managed?

Currently, the majority of available DOAC drug interaction data only illustrate changes in drug exposure conducted in pharmacokinetic studies of healthy volunteers. Available pharmacokinetic drug interaction data in actual patients are limited to subsets of the larger atrial fibrillation population clinical trials. It is unknown if these pharmacokinetic changes translate to pharmacodynamic effect resulting in excess bleeding or thrombotic events.

**Fig. 2:**
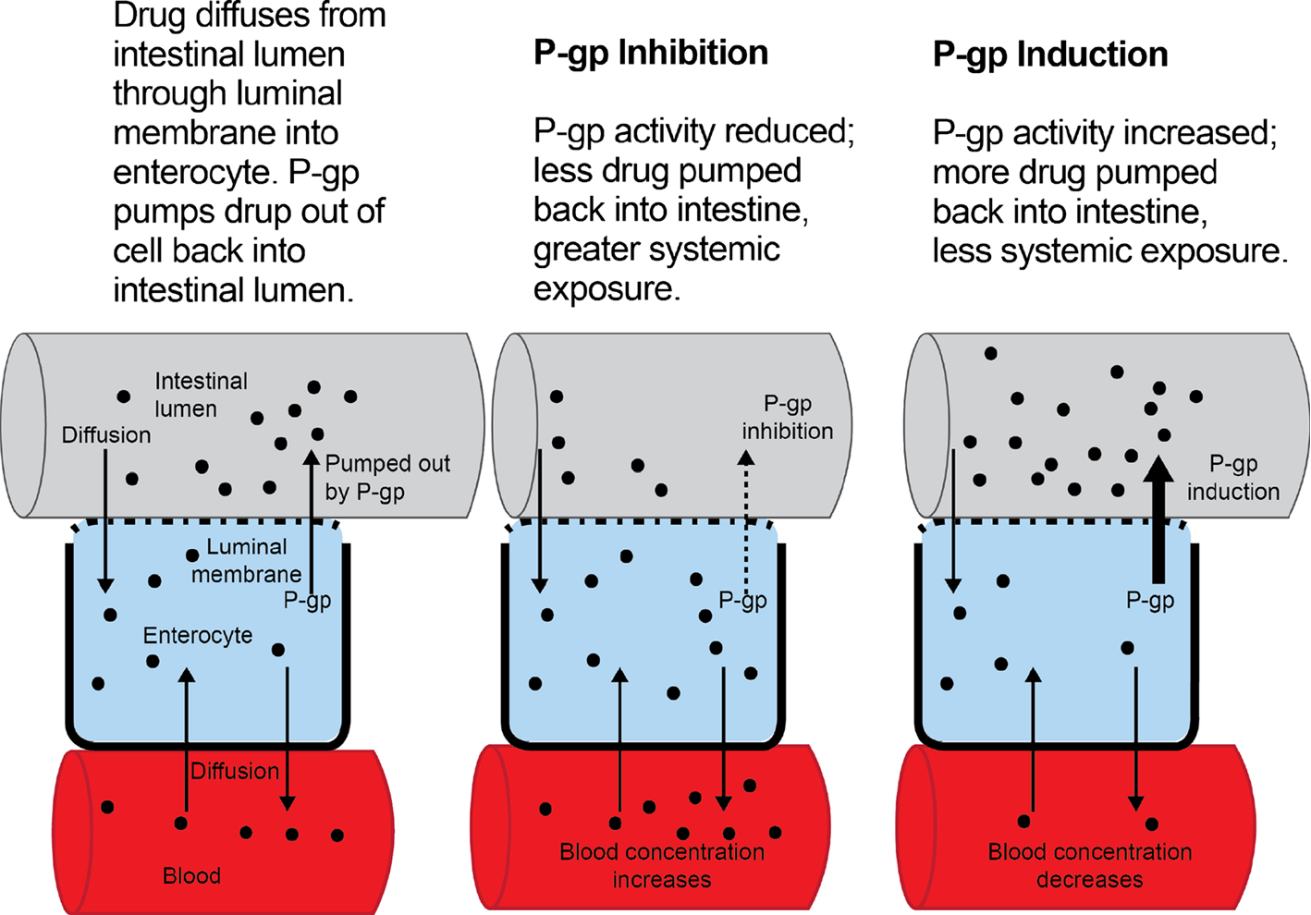
P-gp effect on drug exposure. Reproduced with permission from Kaatz and Mahan [127]

Each of the DOACs is a substrate of permeability-glycoprotein (p-gp), an efflux transporter located in the membranes of the small intestine, blood–brain barrier, liver, and kidneys that regulates absorption of drugs into the bloodstream and tissues [48, 49] (Fig. 2). Hepatic enzyme Cytochrome 3A4 (CYP 3A4) metabolizes rivaroxaban and apixaban to varying degrees (33 and 25 %, respectively). Dabigatran is not a CYP3A4 substrate, and less than 4 % of edoxaban is metabolized via CYP3A4 (Table 12). Drugs that *induce (*increase the function of) p-gp and/or CYP3A4 may decrease DOAC plasma concentrations and increase the risk for thromboembolic events, while drugs that *inhibit* (decrease the function of) p-gp and/or CYP3A4 may increase DOAC concentrations and increase bleeding risk.

**Table 12:** Drug transport/metabolism/elimination characteristics of the direct oral anticoagulants [11, 12, 15, 16, 48, 49, 128, 129]

	P-gp substrate	CYP3A4 substrate (% of drug metabolized via CYP3A4)	% renal elimination
Dabigatran	Yes	No	≈80
Rivaroxaban	Yes	Yes (≈33)^a^	≈33
Apixaban	Yes	Yes (≈25)^b^	≈25
Edoxaban	Yes	No	≈50

*CYP3A4* Cytochrome 3A4, *p-gp* permeability-glycoprotein

^a^Total of ≈66 % hepatic metabolism equally distributed between CYP3A4 and CYP2J2

^b^Total of ≈25 % hepatic metabolism, mostly by CYP3A4, with minor contributions by CYP1A2, 2J2, 2C8, 2C9, and 2C19

Given that each of the DOACs has some proportion of renal elimination (dabigatran 80 %, rivaroxaban 33 %, apixaban 25 %, edoxaban 50 %) [49] (Table 12), patients with renal impairment or over age 75 years taking DOACs may be at a higher risk of bleeding complications [48, 50–56], especially if they also have potential concomitant drug interactions (e.g. taking a p-gp and/or CYP3A4 inhibitor). It is important to note that these same patient characteristics (increasing age, impaired renal function and drug interactions) have been shown to convey an increased bleeding risk with warfarin as well [57].

In VTE treatment trials, dyspepsia and gastrointestinal (GI) bleeding were more common in patients taking dabigatran as compared to warfarin or placebo [6, 7]. Patients with these adverse effects may be frequently prescribed proton-pump inhibitors (PPIs). Even though dabigatran requires an acidic gastric environment for absorption [58], pharmacokinetic studies have not shown a clinically significant reduction in dabigatran exposure with concomitant PPI [14, 59]. Therefore, PPIs may be safely co-administered with dabigatran without need for dose adjustment. Tables 13 and 14 provide an evidence-based summary of drug interactions with dabigatran and the anti-Xa inhibitors. Additionally, the product labeling for each of the DOACs contains detailed dosing information and necessary adjustments that consider route of metabolism and elimination and degree of renal impairment.

**Table 13:** Permeability glycoprotein (p-gp) drug–drug interactions with dabigatran and edoxaban [16, 48, 59, 130–135] (list is not exhaustive)

P-gp inducers	Interacting drug’s effect on dabigatran and edoxaban concentrations	Suggested management
Barbiturates	↓, no specific studies	Avoid use of dabigatran or edoxaban with p-gp *inducers*
Carbamazepine	↓, no specific studies	
Dexamethasone	↓, no specific studies	
Phenytoin	↓, no specific studies	
Rifampin	↓ dabigatran exposure by 66 % ↓ edoxaban exposure	
St John’s Wort	↓, no specific studies	

P-gp inhibitors	Interacting drug’s effect on dabigatran and edoxaban concentrations	Suggested management
Amiodarone	↑, dabigatran exposure by 12-58 % ↑, edoxaban exposure by 40 %	Avoid use of dabigatran with any p-gp *inhibitor* if the patient’s CrCl is < 50 mL/min Reduce edoxaban dose from 60 mg once daily to 30 mg once daily if patient is also taking a p-gp *inhibitor*
Carvedilol	↑, no specific studies	
Clarithromycin	↑, dabigatran exposure by 49 % ↑, no specific studies with edoxaban	
Conivaptan	↑, no specific studies	
Cyclosporine	↑, dabigatran exposure in in vitro studies ↑, edoxaban exposure	
Diltiazem	↑, no specific studies	
Dronedarone	↑, dabigatran exposure by 70–140 % ↑, edoxaban exposure by 85 %	
Erythromycin	↑, no specific studies with dabigatran ↑, edoxaban exposure	
Grapefruit	↑, no specific studies	
Indinavir	↑, no specific studies	
Itraconazole	↑, dabigatran exposure in in vitro studies ↑, no specific studies with edoxaban	
Ketoconazole	↑, dabigatran exposure by 153 % ↑, edoxaban exposure	
Lapatinib	↑, no specific studies	
Mefloquine	↑, no specific studies	
Nelfinavir	↑, dabigatran exposure in in vitro studies ↑, no specific studies with edoxaban	
Nicardipine	↑, no specific studies	
Propafenone	↑, no specific studies	
Quinidine	↑, dabigatran exposure by 53 % ↑, edoxaban exposure by 77 %	
Ritonavir	↑, dabigatran exposure in in vitro studies ↑, no specific studies with edoxaban	
Saquinavir	↑, no specific studies	
Tacrolimus	↑, dabigatran exposure in in vitro studies ↑, no specific studies with edoxaban	
Tamoxifen	↑, no specific studies	
Verapamil	↑, dabigatran exposure by 23–54 % ↑, edoxaban exposure by 53 %	

*CrCl* Creatinine clearance, *p-gp* permeability glycoprotein

**Table 14:** Permeability glycoprotein (p-gp) and Cytochrome 3A4 drug–drug Interactions with rivaroxaban and apixaban) [134–139] (list is not exhaustive)

P-gp and *strong* CYP3A4 inducers	Interacting drug’s effect on rivaroxaban/apixaban concentration	Suggested management
Barbiturate	↓, no specific studies	Avoid use of rivaroxaban or apixaban with p-gp and strong CYP3A4 *inducers*
Carbamazepine	↓, no specific studies	
Phenytoin	↓, no specific studies	
Rifampin	↓, rivaroxaban and apixaban exposure by 50 %	
St John’s Wort	↓, no specific studies	

P-gp and *strong* CYP3A4 inhibitors	Interacting drug’s effect on Factor Xa inhibitor concentration	Suggested management
Clarithromycin	↑, rivaroxaban exposure by 54 % ↑,no specific studies for apixaban	Rivaroxaban: Avoid use of rivaroxaban with p-gp and strong CYP3A4 *inhibitors* Apixaban: If taking 5 mg or 10 mg BID reduce dose by 50 % if combined with strong p-gp and CYP3A4 *inhibitors* If taking 2.5 mg BID avoid apixaban with strong p-gp and CYP3A4 *inhibitors*
Conivaptan	↑, no specific studies	
Grapefruit	↑, no specific studies	
Indinavir	↑, no specific studies	
Itraconazole	↑, no specific studies	
Ketoconazole	↑, rivaroxaban exposure by 160 %	
	↑, apixaban exposure by 200 %	
Nelfinavir	↑, no specific studies	
Posaconazole	↑, no specific studies	
Ritonavir	↑, rivaroxaban exposure by 160 % ↑, no specific studies for apixaban	
Saquinavir	↑, no specific studies	

P-gp and *moderate* CYP3A4 inhibitors	Interacting drug’s effect on rivaroxaban/apixaban concentration	Suggested management
Cyclosporine	↑, no specific studies	Rivaroxaban: Avoid use of rivaroxaban with p-gp and moderate CYP3A4 inhibitors if CrCl is < 80 mL/min Apixaban: No dose adjustment is recommended with p-gp and *moderate* CYP3A4 inhibitors. Use with caution
Diltiazem	↑, apixaban exposure by 30–40 % ↑, no specific studies with rivaroxaban	
Dronedarone	↑, no specific studies	
Tamoxifen	↑, no specific studies	
Verapamil	↑, no specific studies	

*CrCl* Creatinine clearance, *CYP3A4* cytochrome 3A4, *p-gp* permeability lycoprotein

*Antiplatelet agents and non-steroidal anti-inflammatory drugs (NSAIDs)*

When each of the DOACs were studied in combination with dual antiplatelet therapy (aspirin and clopidogrel) for acute coronary syndromes, investigators observed a clinically significant increase in major bleeding in patients taking triple therapy [60–62]. The DOAC VTE treatment trials permitted low-dose concomitant aspirin, and dual antiplatelet therapy was permitted in the dabigatran and rivaroxaban trials. The rate of low-dose aspirin use in the study populations for dabigatran, rivaroxaban, and apixaban ranged from 8 to 14 % and was not reported in the edoxaban trial [4, 5, 7–10, 63]. In a sub-analysis of the rivaroxaban VTE treatment trial, patients taking rivaroxaban and low-dose aspirin had a significantly higher risk of clinically relevant bleeding (hazard ratio (HR) 1.81, 95 % CI 1.36–2.41) and a non-significant increase in major bleeding (HR 1.50, 95 % CI 0.63–3.61) compared to rivaroxaban-only patients [63].

Each of the VTE treatment trials allowed concomitant NSAID use (the edoxaban trial restricted NSAID use to <4 days per week [4, 5, 7–10], with 43 % of dabigatran patients and 23 % of rivaroxaban patients reporting concomitant NSAID use) [7, 60]. Patients taking rivaroxaban and NSAIDs had a 2.5-fold higher rate of major bleeding (HR 2.56, 95 % CI 1.21–5.39) and a 2-fold higher rate of clinically relevant bleeding (HR 1.9, 95 % CI 1.45–2.49) compared to those not taking NSAIDs. In this study, 14 % of the clinically relevant bleeding events were gastrointestinal [63].

Potential drug interactions should be assessed to determine if an alternative non-interacting medication is available to treat the patient’s condition. The duration of interaction exposure should be evaluated, as well as the patient’s risk for a recurrent VTE or major bleeding. Patients at high risk of recurrent VTE (VTE event in the last 3 months or with ongoing VTE risk factors) or at a high risk of bleeding may be particularly vulnerable to DOAC drug interactions. Conversely, patients at a lower risk of recurrent VTE or bleeding may be able to tolerate a moderate drug–drug interaction combination without substantially increasing their risk of adverse events. As always, it is imperative to educate and involve the patient in the discussion. If there is no clear guidance from the literature regarding a specific drug interaction, explain this to the patient and the potential risks involved of each possible approach to management, including alternate therapy. Regular follow-up is advised to assess for adverse events.

### **Guidance statement**

*DOAC drug–drug interaction management must be patient-specific and incorporate multiple clinical parameters, such as concomitant renal impairment, extremes of body weight or advanced age. We suggest that clinicians avoid concomitant use of dabigatran and edoxaban with a strong inducer or inhibitor of p-gp and avoid use of rivaroxaban and apixaban with combined strong inducers and inhibitors of p-gp and CYP3A4.*

*For patients requiring concomitant DOAC therapy with a p-gp and/or CYP3A4 inhibitor, we suggest clinicians closely follow the detailed dose adjustments or avoidance provided in the product labeling. We suggest concomitant antiplatelet or NSAIDs be avoided during DOAC therapy unless the potential benefit clearly justifies the increased bleeding risk.*

6. How should patients transition between anticoagulants?

In general, the need to switch between agents exposes the patient to periods of increased thromboembolic and bleeding risks. In the ROCKET AF [64] and ARISTOTLE trials [65] of rivaroxaban and apixaban, respectively, a 4-fold increase risk of stroke or bleeding was seen at the end of the study period, attributable to lack of a structured approach to ensuring study patients did not have a “gap” in therapeutic levels of anticoagulation while transitioning to warfarin [66]. This underscores the importance of having a carefully constructed and thoughtful approach for anticoagulant transitions, especially for transition to warfarin.

A recent study from a large outpatient anticoagulation clinic showed approximately 4–6 % of their warfarin patients are being switched to a DOAC annually [67]. A Danish study among atrial fibrillation patients found that the majority (51.2 %) of patients prescribed a DOAC had switched to a VKA within 6 months. Reasons for the high rate of switching in this study are not known. However, these two studies collectively suggest that switches between anticoagulants are not infrequent and may be expected to increase [68].

There are a variety of reasons patients may switch between anticoagulants [66]. Patients may require a switch from parenteral anticoagulants to DOAC for longer-term outpatient management. Patients may also be switched from warfarin to a DOAC, or DOAC to DOAC, if they experience a therapeutic failure, have drug intolerance (e.g. rash, dyspepsia, etc.) or if they express a preference for DOAC therapy and are deemed to be an appropriate candidate based on criteria previously discussed [66].

In addition, there may be times when a patient needs to be switched from a DOAC to warfarin, for many of the same reasons, such as drug intolerance, failure or preference. Patients may also acquire a new condition or comorbidity that is a contraindication to DOAC therapy, such as pregnancy, severe renal impairment, placement of a mechanical valve or need for dual antiplatelet therapy that necessitates a switch [66].

Other situations that might warrant a switch include gastric bypass surgery where gastric absorption may be significantly altered or the need for new medication, such as protease inhibitor, that poses a major drug interaction with a DOAC. In these instances it may be best to maintain the patient on warfarin therapy so levels of anticoagulation can be readily monitored. Patients may also not be able to tolerate oral medications during the perioperative period (e.g. bowel resection or NPO status) and thus may need to be transitioned from a parenteral back to a DOAC or from prophylactic-dose DOAC to treatment-dose DOAC [66].

If a VTE patient requires a switch between anticoagulants, clinicians should employ a carefully constructed approach that takes into consideration the patient’s anticoagulation status at the time of the switch, their renal function and the pharmacokinetics of the individual DOAC to avoid significant under- or over anticoagulation of their patient.

Tables 15 and 16 provide information regarding appropriate switching strategies for heparin, LMWH and the DOACs.

**Table 15:** Switching to DOACs

Warfarin to DOAC	
Dabigatran^a^	Start when INR < 2.0
Rivaroxaban^a^	Start when INR < 3.0
Apixaban^a^	Start when INR < 2.0
Edoxaban^a^	Start when INR ≤ 2.5
LMWH to DOAC	
Dabigatran	
Rivaroxaban	Start DOAC within 0–2 h of the time of next scheduled dose of LMWH
Apixaban	
Edoxaban	
(iv) UFH to DOAC	
Dabigatran^a^	
Rivaroxaban^a^	Start DOAC immediately after stopping iv UFH
Apixaban^a^	
Edoxaban^a^	Start edoxaban 4 h after stopping iv UFH

As a general rule, we suggest that as INR drops below 2.5, a DOAC can be started

As a general rule, we suggest that each DOAC can be started within 30 min after stopping (iv) UFH

^a^Recommendations adapted from company’s package insert

**Table 16:** Switching to warfarin

DOAC to warfarin	
Dabigatran^a^	Start warfarin and overlap with dabigatran; CrCl ≥50 mL/min, overlap 3 days CrCl 30–50 mL/min, overlap 2 days CrCl 15–30 mL/min, overlap 1 day
Rivaroxaban^a^ Apixaban^a^	Stop DOAC; start warfarin and LMWH at time of next scheduled DOAC dose and bridge until INR ≥ 2.0
Edoxaban^a^	For 60 mg dose, reduce dose to 30 mg and start warfarin concomitantly For 30 mg dose reduce dose to 15 mg and start warfarin concomitantly Stop edoxaban when INR ≥ 2.0

Overlap intended to avoid under-anticoagulation while warfarin effect developing. When DOAC overlapped with warfarin, measure INR just before next DOAC dose since DOAC can influence INR

As a general rule, we believe either approach (i.e. stop DOAC then start LMWH and warfarin; or overlap warfarin with DOAC, measure INR just before next DOAC dose and stop DOAC when INR ≥ 2.0) can be used for all DOAC to warfarin transitions

*CrCl* creatinine clearance

^a^Recommendations adapted from company’s package insert

### **Guidance statement**

*Switching from warfarin to a DOAC:*

*When switchingfromwarfarin to dabigatran, apixaban, rivaroxaban or edoxaban, discontinue warfarin and start the DOAC when the International Normalized Ratio (INR) has decreased to <2 for dabigatran and apixaban (<3 for rivaroxaban, <2.5 for edoxaban) to avoid periods of inadequate or excessive anticoagulation. In cases where the target INR was 2.5–3.5 or higher due to recurrent VTE, initiate the DOAC when the INR is near 2.5 or the lower end of the specified range.*

*Switching from non-warfarin anticoagulant to a DOAC:*

*When switchingfrom a DOAC to a different DOAC orfrom LMWH/fondaparinux to a DOAC, start the new DOAC 0–2 h prior to the next scheduled administration of the original anticoagulant and then discontinue the original anticoagulant.*

*When switchingfrom IV UFH to a DOAC, stop the heparin infusion and begin administration of the DOAC at the time of UFH discontinuation.*

*When switchingfrom SC UFH treatment to a DOAC, stop the SC UFH and initiate the DOAC approximately 4–5 h after the last dose of SC UFH.*

For additional information regarding switching from a DOAC to warfarin or a non-warfarin anticoagulant, readers are also referred to the respective chapters within this compendium by Wittkowsky and Witt.

7. How should DOAC-associated bleeding be managed?

In both VTE treatment trials and atrial fibrillation trials, rates of major bleeding were shown to be comparable or lower with DOACs than with conventional approaches using LMWH and warfarin [3–10, 64, 65, 69, 70]. There is encouraging evidence to suggest that DOAC patients who develop a major bleed require less blood or factor products, have shorter lengths of hospital stay and potentially have better outcomes compared to patients experiencing VKA-associated major hemorrhage [71–73]. Despite early concerns regarding excessive bleeding with dabigatran, post-marketing surveillance data from the FDA supports a favorable risk–benefit profile [74].

**Fig. 3:**
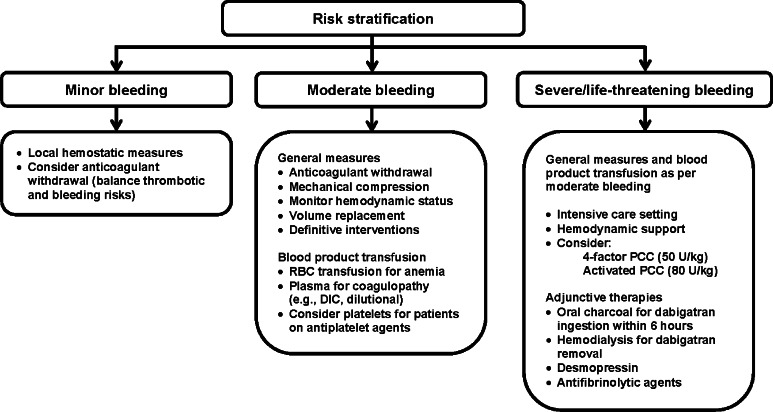
Management of DOAC-associated bleeding

Nevertheless, DOAC-treated patients may experience a hemorrhagic episode and require intervention (Fig. 3). Hospitals should develop evidence-based antithrombotic bleeding and reversal protocols that contain clinical decision support for providers and are easy to access and use in high-stress urgent or emergent situations. The general approach to a bleeding patient, regardless of anticoagulant, includes withholding the anticoagulant, hemodynamic monitoring, resuscitation with fluid and blood products, mechanical compression if possible, and definitive procedural intervention to identify and treat the source of bleed if indicated. In addition to supporting blood pressure, assertive fluid resuscitation will promote renal elimination of DOACs, particularly dabigatran. If DOAC ingestion within the last 6 h can be confirmed, clinicians may consider use of oral activated charcoal for any of the DOACs. In addition to determining time of last DOAC ingestion, clinicians should also rapidly evaluate the patient’s renal function to estimate remaining duration of drug exposure, and potential need for additional interventions, such as hemodialysis. Hemodialysis may be considered for dabigatran patients, particularly if they have impaired renal function and will have prolonged exposure to dabigatran without the aid of extracorporeal removal. Hemodialysis is not an effective option for removal of direct Xa inhibitors due to their extensive protein binding.

If a patient is refractory to general approaches, clinicians may consider non-specific reversal strategies. Several studies of clotting factor concentrates, such as activated and non-activated prothrombin complex concentrates (PCCs) or recombinant Factor VIIa, for DOAC reversal have been reported. This evidence, recently summarized in a systematic review, is of very low quality, as it is limited to in vitro studies, animal models or studies in healthy human volunteers and often shows conflicting results [75]. Additionally, most of these studies evaluated surrogate outcomes, such as normalization of global coagulation assays, instead of relevant clinical outcomes of in vivo hemostasis and mortality. Overall, results suggest that either inactive 4-Factor PCC (KCentra^®^) 50 U/kg or active PCC (aPCC, FEIBA^®^) 80 U/kg are reasonable options for reversal of direct Xa inhibitors and direct thrombin inhibitors, respectively [76–85]. These agents contain procoagulant factors II, VII, IX and X. Activated PCC may pose a greater risk of thrombosis, but may be considered if inactive 4-Factor PCC is not available. Recombinant Factor VIIa is not recommended as a first-line reversal agent. Unlike the PCCs, rFVIIa is not formulated with marginal amounts of anticoagulants (e.g. Protein C, Protein S, Antithrombin, heparin) to mitigate thrombotic risk. Meta-analyses suggest that use of rFVIIa results in higher rates of thrombosis than PCCs [86, 87]. Additionally, because both inactive and active PCCs already contain FVII, there is no rationale to employ rFVIIa as a first-line agent for DOAC reversal. Therefore, we suggest rFVIIa only be used in event PCCs have failed to restore hemostasis in a patient with life-threatening bleeding. Clinicians should carefully weigh risk versus benefit of factor concentrate administration as there is no evidence that these agents improve outcomes and the risk of thrombosis is quite significant [86, 87]. Given the low quality of evidence, it is not unreasonable to withhold these strategies, particularly if there is significant underlying thromboembolic risk.

Fresh frozen plasma should not be used for DOAC reversal, as the volume that would be required to overwhelm the inhibition of thrombin or Factor Xa precludes use in urgent or emergent situations and would likely lead to adverse events, such as fluid overload. Desmospressin or platelet transfusion may be considered in DOAC patients recently on concomitant antiplatelet therapy. Antifibrinolytics agents (tranexamic acid, aminocaproic acid) may be considered as adjunctive therapies if the patient is failing to respond.

Until more robust data or specific antidotes are available, clinicians are limited to existing approaches that have been summarized in several recent reviews [75, 88–90].

Several clinical trials of specific antidotes for both DTIs and Xa inhibitors have been completed or are underway [91–104]. Phase II studies and preliminary data from Phase III studies show these agents to be safe and effective in providing complete and sustained DOAC reversal. They have received expedited review from the FDA and are expected to be commercially available within the next few years.

### **Guidance statement**

*We suggest hospitals develop evidence-based antithrombotic reversal and bleeding protocols that contain clinical decision support for providers and are easy to access and use in urgent or emergent situations. We suggest that general approaches to bleed management be employed for all patients presenting with severe hemorrhage. For DOAC patients, clinicians should attempt to rapidly determine time of last DOAC ingestion and patient’s renal function to estimate remaining duration of exposure and potential utility of additional interventions. Until specific antidotes are available, we suggest clinicians consider use of non-specific reversal strategies in patient’s refractory to standard therapies. For direct Xa inhibitors, non-activated 4-Factor PCC 50 U/kg may be considered. For direct thrombin inhibitors, either 4-Factor non-activated PCC 50 U/kg or activated PCC 80 U/kg may be considered. However, it is reasonable to withhold these strategies given the associated thrombosis risk and the low quality of evidence that they are beneficial in this setting.*

8. What is an appropriate care transitions and follow-up strategy for VTE patients on DOAC therapy?

Inadequate care transitions have been implicated in an estimated annual $25–45 billion in wasted healthcare dollars in the US [105]. Thus, the importance of care transitions has been brought to forefront through numerous national quality initiatives that have emerged in recent years. When looking at approaches specific to anticoagulation patients, implementation of pharmacy-directed anticoagulation services (PDAS) has been shown to significantly improve adherence with specified care transition metrics as well as clinical outcomes [106]. Also, PDAS have been shown to improve patient satisfaction with their care, which now has a direct impact on Medicare reimbursement to hospitals [107]. In efforts to further systematize the delivery of anticoagulation care, reduce adverse drug events and improve care transitions in this high-risk population, a recent consensus statement from EHR Task Force of the New York State Anticoagulation Coalition has called for the incorporation of key anticoagulation-related features into existing EHRs or specialized anticoagulation management systems [108].

There are important nuances in the management of DOACs for VTE, and some of these are not well known. Each of the DOACs requires a dose de-escalation or switch from parenteral therapy at a specified time. The importance of this was recently highlighted in an ISMP alert (https://www.ismp.org/newsletters/acutecare/issue.aspx?id=82) in which a patient prescribed rivaroxaban was given both the 15 mg BID and 20 mg once daily prescriptions prior to discharge. The patient erroneously took both the 15 mg tablets and the 20 mg tablets for several days before the error was discovered. This underscores the importance of clinician familiarity with dosing strategies combined with strong infrastructures, educational processes and thorough handoffs that support accurate and timely implementation of these changes to avoid adverse events [109, 110].

Similar to conventional therapies for VTE treatment, clinicians should evaluate patient eligibility for outpatient or early discharge DOAC therapy, as this has been shown to be safe and effective and provides significant cost savings to the healthcare system [111]. The advent of LMWH and fondaparinux significantly enhanced the feasibility of outpatient treatment during transition to warfarin. Outpatient VTE treatment is made even more feasible with the availability of the DOACs. As with conventional therapies, DOAC patients must meet certain clinical, behavioral and social criteria to be considered a viable candidate for outpatient therapy [111]. For stable patients with acute DVT that does not warrant thrombolysis or thrombectomy, outpatient therapy is an option as long as they are deemed likely to be adherent with medications and follow up, have confirmed ability to obtain the anticoagulant(s), have expressed understanding of their condition and what to do in the event of bleeding or clotting, and have a good social support system at home. Clinicians tend to be less comfortable treating patients with a pulmonary embolism in the outpatient setting. However, evidence for this strategy in appropriately selected patients is increasing. There are clinical prediction tools, such as the modified Pulmonary Embolism Severity Index (PESI) score [112] that aid in identifying PE patients with a low risk of adverse outcomes that may be considered for outpatient treatment (Table 17).

**Table 17:** Simplified PESI (Pulmonary Embolism Severity Index) score [112]

Predicts 30-day outcomes of patients with PE	
Variable	Score
Age >80 years	1
History of cancer	1
History of chronic cardiopulmonary disease	1
Systolic blood pressure <100 mm Hg	1
Heart rate >110	1
O_2_ saturation <90 %	1

Score of 0 = low risk (consider outpatient therapy)

Score >0 = high risk

Care transitions can also occur within the hospital, such as when patients transfer to or from the ICU. At each transition, a review of the patient’s medication profile and communication of therapeutic plans for each patient issue should be affected between the previous and current multidisciplinary teams. Surgical patients on DOACs warrant particular attention during care transitions within the hospital, as clinicians have far more experience with managing temporary interruptions in warfarin therapy, and staff may not be familiar with management of DOACs in the perioperative period, or even recognize DOACs as anticoagulants. Thus, potential transitions between drug therapies and across care settings (e.g. medical ward to OR and back) require thoughtful consideration and planning.

While DOACs do not require routine outpatient monitoring and adjustment, a standardized follow-up strategy needs to be delineated to facilitate periodic patient evaluation for clinically relevant issues [110, 113].

### **Guidance statement**

*We suggest that hospitals implement systematic DOAC management and documentation processes that address appropriate patient selection, dose initiation, perioperative management, switches between anticoagulants and transitions between care settings. Whenever possible, implementation of a specialized inpatient and outpatient anticoagulation services is strongly encouraged. We also strongly recommend that clinicians utilize a DOAC discharge checklist (Table *18*) to ensure all key aspects of patient care and DOAC therapy are addressed.*

**Table 18:** DOAC discharge checklist for optimal care transitions

Patient is an appropriate DOAC candidate
Assess patient’s eligibility for outpatient treatment
Consistent access to DOAC (affordability, retail availability)
If transitioning to rehabilitation or skilled nursing facility, ensure DOAC on formulary
DOAC identified and understood as an oral anticoagulant by patient, caregivers and providers
Provision of thorough DOAC education to patient and/or caregiver in their preferred language and at an appropriate literacy level
Safety net phone number provided to patient/caregiver (Who to call with questions)
Referral or handoff to appropriate provider (anticoagulation clinic, PCP, etc.)
Time of last drug administration in current setting and time of next scheduled dose in new setting
Prescribed strategy for appropriate dose change after initial therapy (either switch to DOAC or DOAC dose de-escalation)
Consolidated documentation and communication to next care setting of key information such as
Indication for anticoagulation
Intended duration of therapy
DOAC dose and scheduled time of administration
Contact information for anticoagulation provider
Follow-up arranged for periodic (every 3–12 months) assessment of the following
Renal function
Liver function
Upcoming invasive procedures
New drug interactions
New contraindications

*DOAC* direct-acting oral anticoagulant, *PCP* primary care physician

9. How can patients enhance safety and efficacy of their DOAC therapy?

Studies have shown that patients who are actively engaged in their healthcare experience have better care experiences, improved outcomes and lower overall healthcare costs [114, 115]. One method to “activate” patients and caregivers is to increase their health literacy via education about their disease state and medication therapies. DOAC education for patients and caregivers should be individualized, drug specific and provided in the patient’s preferred language at an appropriate literacy level. As the number of indications and evidence for DOACs expands, educational tools can quickly become outdated. It is important to involve anticoagulation resources, such as a PDAS, in regularly updating DOAC educational materials or obtaining them from contemporary, reliable sources (Tables 19, 20, and 21).

**Table 19:** Patient education resources

Web-based patient and family educational resources	
Patient Guides published by manufacturer (accompanies Product Insert)	www.pradaxa.com www.xarelto-us.com www.eliquis.com http://www.savaysa.com
Agency for Healthcare Research and Quality (ARHQ) This is specific to warfarin. However general patient safety and disease-specific information is helpful	*Patient booklet* Your guide to preventing and treating blood clots http://www.ahrq.gov/patients-consumers/prevention/disease/bloodclots.html *Patient education video* Blood thinner pills: your guide to using them safely http://www.ahrq.gov/patients-consumers/diagnosis-treatment/treatments/btpills/btpills.html
Anticoagulation forum—Centers of Excellence Resource Center/Patient and Family Education Pillar	http://excellence.acforum.org/

**Table 20:** Drug-specific educational points for DOACs and VTE treatment [11, 12, 15, 16]

Patient and family educational needs				
Warfarin	Dabigatran	Rivaroxaban	Apixaban	Edoxaban
Daily, dose-adjusted	Twice daily	Daily (initially twice daily)	Twice daily	Daily
	Various dose adjustments recommended based on indications, kidney or liver function, and/or concomitant drugs			
Missed dose	Missed dose: take as soon as possible on the same day but 6 h before next scheduled dose	If missed a 15 mg dose, can take 30 mg one time to make up	Take as soon as possible same day	Take as soon as possible on the same day
Take if before midnight on same day				
Call warfarin manager				
Do not double up to make up for missed dose				
+/− food	Take with full glass of water, +/− food	Take with food	+/− food	+/− food
Weekly pill planner can aid compliance	MUST store in original container, keep sealed, use capsules in 120 days	Weekly pill planner can aid compliance		
Can crush, mix with food	Swallow whole, do NOT cut, open, or crush	Can crush and give via NG or gastric tube or mix with food	Can crush, suspend in D5 W and give via NG tube	No data regarding crushing, so crushing not recommended
Numerous drug:drug interactions, report all to warfarin manager	Important drug:drug interactions: P-gp inducers and inhibitors (especially if renal function compromised)	Avoid dual P-gp and strong CYP 3A4 inducers or inhibitors	Avoid dual P-gp and strong CYP 3A4 inducers or inhibitors	Important drug:drug interactions: P-gp inducers and inhibitors
Inform provider of all medication changes, including over-the-counter and herbals				
Carry “anticoagulant ID wallet card” to alert emergency medical responders				
DO NOT stop taking without a physician order (get prescriptions refilled on time)				
Report signs and symptoms of bleeding and/or potential clotting				
Inform all health care providers before invasive procedures or surgery, including dental				
Inform health care provider if pregnant or plan to become pregnant				
Inform health care provider if breastfeeding				
Careful planning and communication around transition of care episodes				

*DOAC* direct oral anticoagulant, *VTE* venous thromboembolism, *NG* nasogastric

**Table 21:** Patient education and safety tips to optimize DOAC use

Suggested patient action	Comment
Ask questions and express your values and preferences in regards to your anticoagulant therapy	Consider all of the possible advantages and disadvantages of DOAC therapy and choose an anticoagulation regimen that you are most likely to be adherent with
Make sure you are familiar with and understand the DOAC education provided to you by healthcare staff	If there is something you do not understand or that concerns you, let the healthcare staff know as soon as possible Have the healthcare provider give you a safety net phone number to call in case you have questions at a later time
Obtain and wear a Medic Alert bracelet or carry a wallet card stating you are on anticoagulant	This will notify medical personnel that you are on an anticoagulant in case you are unable to verbally tell them
Follow drug-specific administration and storage recommendations provided to you	e.g. take with food, store in original container, etc.
Establish a set time for taking your DOAC and communicate this to medical providers, especially in an emergency situations	
Schedule follow-up phone calls with your anticoagulation provider at pre-determined times to discuss any issues or difficulties in taking or refilling your DOAC	
Make sure you are familiar with both the generic and brand names of your DOAC and always check your refill for accuracy before leaving the pharmacy	
Make sure your anticoagulation provider or another provider is regularly checking your kidney and liver function to make sure it is still okay for you to take a DOAC	If you develop kidney or liver problems, let your anticoagulation provider know as soon as possible
Go to or participate in all scheduled follow-up visits with your anticoagulation provider so they can ask you questions that might be important for safe and effective use of your DOAC	What medications have you stopped/started? What kidney/liver problems have you had? What side effects have you had from your DOAC? What problems have you had getting your DOAC refilled? What extra or missed doses of your DOAC have you had? What upcoming surgical or dental procedures do you have?

*DOAC* Direct oral anticoagulant

It is recommended to employ multiple modalities of education, such as verbal, written and video to reinforce key points as this will help achieve better outcomes [116]. Unfortunately, this is not always done. According to a survey conducted by the ISMP [117], 1 in 4 nurses indicate they do not provide written information to accompany verbal information provided to patients about their medications. Common reasons cited included no written materials being available, written materials not available in languages other than English, or written materials not appropriate for patients with poor literacy skills. Written materials should be developed to provide helpful reinforcement and reminders of safety issues.

Understanding how patients prefer to learn, type of media they value most and determining in advance how visual or hearing impairments may impact the educational process will help determine the best educational approach.

As education is provided, confirmation of a patient’s comprehension of their disease and care plan is key. The teachback method (can the patient/caregiver accurately explain the information back to the educating clinician using their own words?) is a widely accepted means of assessing comprehension and should be integrated into all DOAC educational efforts. Including family members, caregivers or significant others in the education process may improve patient care and outcomes.

Table 5 summarizes key characteristics specific to each DOAC that are relevant to optimal use of these agents. These characteristics should be incorporated into comprehensive DOAC patient education processes and should be considered prior to prescribing.

Patients and caregivers are also more actively engaged when their values and preferences are considered. Developing an appreciation for patients’ values and preferences is important to determine the best drug therapy option for them and requires a thoughtful, thorough discussion. The DOACs have many advantages and disadvantages (Table 2) that should be reviewed with patients. For example, no requirement for lab monitoring may be perceived as highly beneficial but there may be significant concern about lack of an antidote. Each of these points should be presented to the patient and/or caregiver for consideration, as it may not only influence the choice of anticoagulant, but also adherence to therapy and clinical outcomes [118].

### **Guidance statement**

*We suggest use of a comprehensive, multi-media educational approach with patients and families to maximize the efficacy and safety associated with anticoagulation in the VTE population. Information should be provided in the patient’s preferred language and at an appropriate level of health literacy.*

## Conclusion

The arrival of the DOACs has rapidly expanded VTE treatment options over the span of just a few years. While barriers remain for specific segments of the VTE population, the DOACs offer treatment options that are not only more convenient, but likely safer than conventional therapy. Although the DOACs represent a significant advance in VTE treatment, complexity of DOAC dosing regimens, potential for drug interactions, and variable effects on commonly available coagulation assays demand expertise from the prescribing clinician and effective patient education to ensure optimal outcomes for patients treated with DOACs for VTE. Table 22 summarizes these guidance statements.

**Table 22:** Summary of guidance statements

Question	Guidance statement
Which VTE patients are (and are not) good candidates for DOAC therapy?	DOACs are suggested as an alternative to conventional therapy for VTE treatment in patients who meet appropriate patient selection criteria. For all other patients, we suggest VTE treatment with conventional therapy. Until further data are available, we suggest avoiding DOACs for VTE in patients with antiphospholipid antibody syndrome and patients at extremes of weight. LMWH monotherapy remains first line for patients with cancer-related VTE, but DOACs may be considered in select patients unwilling or unable to receive subcutaneous injections
How should DOACs be initiated for VTE treatment?	We suggest that a thorough patient evaluation be conducted prior to DOAC initiation which should include assessment of baseline laboratory values, concomitant drug therapies, and comorbidities. We do not recommend initial DOAC therapy in patients who are hospitalized with extensive DVT or who have PE with hemodynamic instability in whom thrombolysis or thrombectomy may be indicated. We suggest that the unique characteristics of each DOAC, their distinct dosing for VTE treatment, and patient preferences should be considered when selecting a DOAC for VTE treatment
How the anticoagulant activity of DOACs be measured?	We suggest that clinicians do not routinely measure DOAC activity. If measurement of a DOAC is indicated, we suggest that clinicians use assays that are validated either locally or in a reference laboratory and that are readily available. The chosen assay should be suitable for the DOAC being used, as well as for the indication for measurement, as detailed in Table 6
How should VTE patients who require temporary interruption of DOAC therapy be managed?	For VTE patients on DOAC therapy requiring TI for an invasive procedure, we suggest a carefully constructed, thoughtful approach that emphasizes communication between the provider managing the DOAC therapy, the clinician performing the procedure, and the patient and/or caregiver about the management of the DOAC. If TI is deemed necessary, we suggest that clinicians consider the patient’s renal function, the DOAC t_1/2_ and the associated bleeding risk when determining timing of cessation and resumption of the DOAC. We suggest avoiding routine use of bridge therapy during DOAC interruption
How should patients with DOAC drug–drug interactions be managed?	DOAC drug–drug interaction management must be patient-specific and incorporate multiple clinical parameters, such as concomitant renal impairment, extremes of body weight or advanced age. We suggest that clinicians avoid concomitant use of dabigatran and edoxaban with a strong inducer or inhibitor of p-gp and avoid use of rivaroxaban and apixaban with combined strong inducers and inhibitors of p-gp and CYP3A4 For patients requiring concomitant DOAC therapy with a p-gp and/or CYP3A4 inhibitor, we suggest clinicians closely follow the detailed dose adjustments or avoidance provided in the product labeling. We suggest concomitant antiplatelet or NSAIDs be avoided during DOAC therapy unless the potential benefit clearly justifies the increased bleeding risk
How should patients transition between anticoagulants?	*Switching from warfarin to a DOAC* When switching *from* warfarin to dabigatran, apixaban, rivaroxaban or edoxaban, discontinue warfarin and start the DOAC when the International Normalized Ratio (INR) has decreased to <2 for dabigatran and apixaban (<3 for rivaroxaban, <2.5 for edoxaban) to avoid periods of inadequate or excessive anticoagulation. In cases where the target INR was 2.5–3.5 or higher due to recurrent VTE, initiate the DOAC when the INR is near 2.5 or the lower end of the specified range *Switching from non-warfarin anticoagulant to a DOAC* When switching *from* a DOAC to a different DOAC or *from* LMWH/fondaparinux to a DOAC, start the new DOAC 0–2 h prior to the next scheduled administration of the original anticoagulant and then discontinue the original anticoagulant When switching *from* IV UFH to a DOAC, stop the heparin infusion and begin administration of the DOAC at the time of UFH discontinuation When switching *from* SC UFH treatment to a DOAC, stop the SC UFH and initiate the DOAC approximately 4–5 h after the last dose of SC UFH
How should DOAC-associated bleeding be managed?	We suggest hospitals develop evidence-based antithrombotic reversal and bleeding protocols that contain clinical decision support for providers and are easy to access and use in urgent or emergent situations. We suggest that general approaches to bleed management be employed for all patients presenting with severe hemorrhage. For DOAC patients, clinicians should attempt to rapidly determine time of last DOAC ingestion and patient’s renal function to estimate remaining duration of exposure and potential utility of additional interventions. Until specific antidotes are available, we suggest clinicians consider use of non-specific reversal strategies in patient’s refractory to standard therapies. For direct Xa inhibitors, non-activated 4-Factor PCC 50 units/kg may be considered. For direct thrombin inhibitors, either 4-Factor non-activated PCC 50 U/kg or activated PCC 80 U/kg may be considered. However, it is reasonable to withhold these strategies given the associated thrombosis risk and the low quality of evidence that they are beneficial in this setting
What is an appropriate care transitions and follow-up strategy for VTE patients on DOAC therapy?	We suggest that hospitals implement systematic DOAC management and documentation processes that address appropriate patient selection, dose initiation, perioperative management, switches between anticoagulants and transitions between care settings. Whenever possible, implementation of a specialized inpatient and outpatient anticoagulation services is strongly encouraged. We also strongly recommend that clinicians utilize a DOAC discharge checklist (Table 18) to ensure all key aspects of patient care and DOAC therapy are addressed
How can patients enhance safety and efficacy of their DOAC therapy?	We suggest use of a comprehensive, multi-media educational approach with patients and families to maximize the efficacy and safety associated with anticoagulation in the VTE population. Information should be provided in the patient’s preferred language and at an appropriate level of health literacy
